# Loss of function of Hog1 improves glycerol assimilation in *Saccharomyces cerevisiae*

**DOI:** 10.1007/s11274-023-03696-z

**Published:** 2023-07-21

**Authors:** Masato Sone, Kantawat Navanopparatsakul, Shunsuke Takahashi, Chikara Furusawa, Takashi Hirasawa

**Affiliations:** 1 School of Life Science and Technology, Tokyo Institute of Technology, 4259 Nagatsuta-cho, Midori-ku, Yokohama, Kanagawa 226-8501 Japan; 2 Center for Biosystem Dynamics Research, RIKEN, 6-2-3 Furuedai, Suita, Osaka 565-0874 Japan; 3 Universal Biology Institute, The University of Tokyo, 7-3-1 Hongo, Bunkyo-ku, Tokyo, 113- 0033 Japan; 4 Department of Physics, Graduate School of Science, The University of Tokyo, 7-3-1 Hongo, Bunkyo-ku, Tokyo, 113-0033 Japan

**Keywords:** Glycerol, High osmolarity glycerol pathway, Hog1, L-Lactic acid, *Saccharomyces cerevisiae*

## Abstract

We previously isolated a mutant of *Saccharomyces cerevisiae* strain 85_9 whose glycerol assimilation was improved through adaptive laboratory evolution. To investigate the mechanism for this improved glycerol assimilation, genome resequencing of the 85_9 strain was performed, and the mutations in the open reading frame of *HOG1*, *SIR3*, *SSB2*, and *KGD2* genes were found. Among these, a frameshift mutation in the *HOG1* open reading frame was responsible for the improved glycerol assimilation ability of the 85_9 strain. Moreover, the *HOG1* gene disruption improved glycerol assimilation. As *HOG1* encodes a mitogen-activated protein kinase (MAPK), which is responsible for the signal transduction cascade in response to osmotic stress, namely the high osmolarity glycerol (HOG) pathway, we investigated the effect of the disruption of *PBS2* gene encoding MAPK kinase for Hog1 MAPK on glycerol assimilation, revealing that *PBS2* disruption can increase glycerol assimilation. These results indicate that loss of function of Hog1 improves glycerol assimilation in *S. cerevisiae*. However, single disruption of the *SSK2*, *SSK22* and *STE11* genes encoding protein kinases responsible for Pbs2 phosphorylation in the HOG pathway did not increase glycerol assimilation, while their triple disruption partially improved glycerol assimilation in *S. cerevisiae*. In addition, the *HOG1* frameshift mutation did not improve glycerol assimilation in the *STL1*-overexpressing *RIM15* disruptant strain, which was previously constructed with high glycerol assimilation ability. Furthermore, the effectiveness of the *HOG1* disruptant as a bioproduction host was validated, indicating that the *HOG1 CYB2* double disruptant can produce L-lactic acid from glycerol.

**Supplementary Information:**

The online version contains supplementary material available at 10.1007/s11274-023-03696-z.

## Introduction

The budding yeast *Saccharomyces cerevisiae* has been widely used not only for brewing alcoholic beverages, such as wine and Japanese rice wine (sake), but also for producing bioethanol and industrially useful materials, including organic acids, alcohols, and intermediate compounds of pharmaceutical drugs. Moreover, it has been used as a eukaryotic model organism for researches in genetics and molecular biology.

In biodiesel production, triacylglycerol obtained from plants and animal oils is converted to fatty acid esters and glycerol by transesterification with alcohols (Parawira 2009). Fatty acid esters can be used as fuels, but there are few appropriate ways to utilize glycerol as a byproduct in biodiesel production. Therefore, efficient strategies for using glycerol are highly desirable. To date, many studies on glycerol utilization as a carbon source for bioproduction using microorganisms have been reported (Asskamp et al. 2019; Jo et al. 2023; Tokuyama et al. 2014; Vikromvarasiri et al. 2021; Zhu et al. 2002).

It is known that *S. cerevisiae* has metabolic reactions to assimilate glycerol as a carbon source (Klein et al. 2017). However, *S. cerevisiae* cannot grow well on glycerol as a sole carbon source, and the reason for this phenomenon is unclear (Swinnen et al. 2013). Previously, to obtain *S. cerevisiae* strains with high glycerol assimilation ability through metabolic engineering, we performed adaptive laboratory evolution of the *S. cerevisiae* W303-1B strain, in which the serial transfer of a culture to a fresh medium containing glycerol as the main carbon source was repeated until the cell growth rate increased by three times (Kawai et al. 2019). Based on transcriptome analysis of the evolved population exhibiting high growth rate on glycerol, we constructed recombinant strains with high growth ability on glycerol, and we successfully improved glycerol assimilation by overexpressing *HAP4* or disrupting *RIM15* genes together with overexpressing the *STL1* gene. However, the growth rate of the recombinant strains was lower than that of the population obtained by adaptive laboratory evolution. In addition, the metabolic flux distribution of the evolved 85_9 strain, which was isolated by single colony isolation of the evolved population after 85 generations in the adaptive laboratory evolution, showed the fastest growth rate among the strains isolated (Yuzawa et al. 2021). However, the mechanisms for the improved glycerol assimilation in 85_9 strain are still unclear.

Glycerol functions as a compatible solute against increasing osmotic pressure in *S. cerevisiae* (Blomberg and Adler 1989). When *S. cerevisiae* responds to an increase in osmotic pressure, a signal transduction cascade, namely high osmolarity glycerol (HOG) pathway, is activated, and the mitogen-activated protein kinase (MAPK) Hog1 is phosphorylated (Brewster et al. 1993). Two branches are present in the HOG pathway; Sln1 and Sho1. In the Sln1 branch, the signal is transferred by a phosphorylation relay from Sln1 on the cytoplasmic membrane through Ypd1 and Ssk1 to MAPK kinase kinases (MAPKKKs) Ssk2 and Ssk22 in the cytoplasm (Posas et al. 1996). In the Sho1 branch, the Sho1 protein on the cytoplasmic membrane recruits the Ste11 MAPKKK via a scaffold protein Pbs2, which is a MAPK kinase (MAPKK) responsible for the phosphorylation of Hog1 (Posas and Saito 1997). Phosphorylated Ssk2, Ssk22, and Ste11 phosphorylate Pbs2 MAPKK, and phosphorylated Pbs2 phosphorylates Hog1 MAPK (Brewster et al. 1993; Posas and Saito 1997; Posas et al. 1996). Phosphorylated Hog1 MAPK is transferred to the nucleus with importin β encoded by *NMD5* (Ferrigno et al. 1998) and phosphorylates the stress-responsive transcription factors Msn2 and Msn4 (Rep et al. 2000). Phosphorylated Msn2 and Msn4 induce the transcription of genes related to glycerol synthesis (Rep et al. 2000). However, the relationship between Hog1 and glycerol assimilation remains unknown.

In the present study, we performed genome resequencing analysis of *S. cerevisiae* 85_9 strain and evolved population to understand the mechanisms of glycerol assimilation, and we identified the mutation responsible for improved glycerol assimilation in that strain. We found that loss of function of Hog1 by its mutation or *PBS2* disruption resulted in improved glycerol assimilation in *S. cerevisiae*. Moreover, the effect of disruption of genes related to the HOG pathway on glycerol assimilation was investigated. In addition, the effectiveness of the *HOG1* disruptant as a host for bioproduction from glycerol was demonstrated.

## Materials and methods

### Strains, plasmids, and media

*S. cerevisiae* W303-1B (*MAT*α *leu2-3*,*112 trp1-1 can1-100 ura3-1 ade2-1 his3-11,15*) and its glycerol-assimilating mutant strain 85_9, previously obtained through adaptive laboratory evolution and single-colony isolation (Yuzawa et al. 2021), were used. In addition, the *STL1*-overexpressing *RIM15* disruptant of *S. cerevisiae* constructed in a previous study (Kawai et al. 2019) was used. For recombinant DNA experiments, *Escherichia coli* MG1655 (F^–^, wild-type) and DH5α (F^–^ Φ80d*lacZ*ΔM15 Δ(*lacZYA-argF*) U169 *deoR recA*1 *endA*1 *hsdR*17(r_K_^–^, m_K_^+^) *phoA supE*44 λ^–^*thi*-1 *gyrA*96 *relA*1) were used.

To amplify the gene disruption cassette for *S. cerevisiae*, the plasmid pUG73 carrying the *LEU2* gene from *Kluyveromyces lactis* (*KlLEU2*) (Gueldener et al. 2002) was used. To remove the selection marker in the disruption cassette from the genome of gene disruptants in *S. cerevisiae*, plasmid pSHAUR1 (Ida et al. 2012) was used. To clone the mutant *KGD2* gene from the 85_9 strain and the gene encoding L-lactate dehydrogenase (LDH) from *Xenopus laevis*, a T-vector pMD20 (Takara Bio Inc., Shiga, Japan) and an expression vector for *S. cerevisiae* pGK426 (Ishii et al. 2009), provided by the National Bio-Resource Project, Japan, were used. In addition, pGK425-*STL1*, which was constructed in a previous study (Kawai et al. 2019), was used to overexpress the *STL1* gene in *S. cerevisiae*.

To construct recombinant strains of *S. cerevisiae*, YPAD medium [10 g L^–1^ Bacto yeast extract (Difco Laboratories, Detroit, MI), 20 g L^–1^ Bacto peptone (Difco Laboratories), 20 g L^–1^ glucose, and 0.2 g L^–1^ adenine hemisulfate], 2× YPAD medium (20 g L^–1^ Bacto yeast extract, 40 g L^–1^ Bacto peptone, 40 g L^–1^ glucose, 0.4 g L^–1^ adenine hemisulfate), YPAGal medium (10 g L^–1^ Bacto yeast extract, 20 g L^–1^ Bacto peptone, 20 g L^–1^ galactose, 0.2 g L^–1^ adenine hemisulfate), and SC medium [6.7 g L^–1^ yeast nitrogen base without amino acids (Difco Laboratories), 20 g L^–1^ glucose, 0.2 g L^–1^ adenine hemisulfate, 0.076 g L^–1^ L-tryptophan, 0.076 g L^–1^ uracil, 0.076 g L^–1^ L-leucine, 0.076 g L^–1^ L-histidine-HCl, 1.4 g L^–1^ yeast synthetic drop-out media supplements without histidine, leucine, tryptophan, and uracil (Sigma-Aldrich, Inc., St. Louis, MO)] were used. To prepare agar plates, 20 g L^–1^ agar was added to the medium. To analyze glycerol assimilation in *S. cerevisiae*, MD medium (6.7 g L^–1^ yeast nitrogen base without amino acid, 20 g L^–1^ glucose, 0.2 g L^–1^ adenine hemisulfate, 0.076 g L^–1^ L-histidine-HCl, 0.076 g L^–1^ L-tryptophan, 0.076 g L^–1^ L-leucine, 0.076 g L^–1^ uracil) and MG medium, in which 20 g L^–1^ glycerol was added instead of glucose, were used. For the assay of L-lactate production, modified MG medium containing 6.7 g L^–1^ yeast nitrogen base without amino acid, 20 g L^–1^ glycerol, 0.2 g L^–1^ adenine hemisulfate, 0.38 g L^–1^ L-histidine-HCl, 0.38 g L^–1^ L-tryptophan, 0.38 g L^–1^ L-leucine was used. If necessary, uracil and L-leucine were removed from the culture medium.

For plasmid construction, *E. coli* was cultured in Lennox (L) medium containing 10 g L^–1^ hipolypeptone (Nihon Pharmaceutical Co., Ltd., Tokyo, Japan), 5 g L^–1^ dried yeast extract D-3 H (Nihon Pharmaceutical Co.), 5 g L^–1^ NaCl, and 1 g L^–1^ glucose (pH 7.0). To prepare the L agar plates, 15 g L^–1^ agar was added to the medium. If necessary, 50 mg L^–1^ ampicillin was added to the L medium.

### Genome resequencing analysis

For genome resequencing analysis, genomic DNA was isolated from *S. cerevisiae* using Gentra Puregene Yeast/Bact. kit (Qiagen, Venlo, The Netherlands). Genome resequencing analysis of the *S. cerevisiae* W303-1B and 85_9 strains, as well as the cell population obtained from the culture after 85 generations in the adaptive laboratory evolution, was performed using the MiSeq system (Illumina, Inc., San Diego, CA). Mutations in the 85_9 strain and cell population after 85 generations of adaptive laboratory evolution were identified using the Mudi web tool (https://naoii.nig.ac.jp/mudi_top.html) (Iida et al. 2014).

The fastq files obtained from genome resequencing analysis are available under accession numbers DRA452749, DRA452750, and DRA452751.

### Introduction of mutations identified in the 85_9 mutant into the W303-1B strain

It was reported that the expression of *E. coli mazF* gene, which encodes mRNA interferase, is toxic to *S. cerevisiae* (Liu et al. 2014). Based on this report, we developed a system to introduce mutations into *S. cerevisiae*. Briefly, the *mazF* gene with the *S. cerevisiae GAL1* promoter was first introduced into the target gene site of *S. cerevisiae* grown in a medium containing glucose to prevent expression of the *mazF* gene. The resulting strain was transformed again with the DNA fragment carrying a mutation, and the transformants were selected on a medium containing galactose. If the *mazF* gene in the target site is replaced with the introduced DNA fragment carrying a mutation, cells can grow on galactose.

First, a plasmid containing *KlLEU2* and *E. coli mazF* was constructed. *E. coli mazF* gene was amplified through polymerase chain reaction (PCR) from the genomic DNA of *E. coli* MG1655 using KOD-Plus-Neo (Toyobo Co., Ltd., Osaka, Japan) and the primer set shown in Table S1. In addition, the *GAL1* promoter and *CYC1* terminator were amplified from the genomic DNA of *S. cerevisiae* W303-1B through PCR using the primer sets shown in Table S1. These three fragments were connected using overlap extension PCR, and the connected fragment was inserted into the SacI site of pUG73. The resulting plasmid was designated pUG73-*mazF*.

Next, the DNA fragments containing the *KlLEU2* and *mazF* genes with upstream and downstream flanking sequences of *HOG1*, *SIR3*, and *SSB2* genes were amplified through PCR from the pUG73-*mazF* using primer sets shown in Table S1. Each amplified fragment was introduced into the W303-1B strain using the lithium acetate method reported by Gietz and Woods (2002) and L-leucine-prototrophic transformants were obtained on SC medium. Then, the DNA fragment was amplified from the genomic DNA of the 85_9 strain through PCR using the primer sets shown in Table S1, introduced into the corresponding L-leucine-prototrophic transformant, and transformants were selected on YPAGal medium. The introduction of mutations was confirmed by sequencing of the DNA fragment amplified through PCR from the genome of the transformant selected on YPAGal medium using the primer sets shown in Table S1.

As the strain carrying the *KGD2* gene mutation found in the 85_9 strain could not be obtained using the methods described above, the *KGD2*-disrupted strain harboring a plasmid with the mutant *KGD2* gene was constructed. The mutant *KGD2* gene was amplified from the genomic DNA of the 85_9 strain, through PCR using the primer set shown in Table S1. The amplified fragment was treated with 10×A attachment mix (Toyobo Co.) and cloned into the T-vector pMD20. After confirming the nucleotide sequence of the cloned fragment, it was subcloned into the SalI-EcoRI sites of pGK426 (Ishii et al. 2009). The resulting plasmid was introduced into the *KGD2*-disrupted strain. The methods for constructing the *KGD2*-disrupted strain are described in the following section.

### Gene disruption in *S. cerevisiae*

To disrupt genes in *S. cerevisiae*, a disruption cassette (*loxP*-*KlLEU2*-*loxP*) with upstream and downstream flanking regions for each target gene was amplified through PCR from the template plasmid pUG73 (Gueldener et al. 2002) using KOD-Plus-Neo and KOD One (Toyobo Co.) and primer sets listed in Table S1. The disruption cassettes were introduced into the W303-1B strain, and the disruption of the target genes was checked through PCR using the primer sets shown in Table S1. To disrupt multiple genes, *KlLEU2* sequence at the disrupted site was removed from the genome by introducing plasmid pSHAUR1 (Ida et al. 2012) into the constructed disruptants and expressing Cre recombinase in YPGal medium to induce homologous recombination between *loxP* sites in the disruption cassette, and then a disruption cassette for another gene was introduced.

### Construction of a plasmid carrying the L-lactate dehydrogenase gene from *X. laevis*

To construct a plasmid carrying the gene for LDH from *X. laevis*, a gene fragment, whose codons were optimized for *S. cerevisiae*, was synthesized (GeneArt Strings DNA Fragments; Thermo Fischer Scientific, Waltham, MA) (Fig. S1). The fragment was amplified through PCR from this synthesized fragment using the primer set shown in Table S1 and then cloned into pMD20. After confirming the nucleotide sequence of the cloned fragment, it was subcloned into the SalI-BamHI sites of pGK426 (Ishii et al. 2009), and the resulting plasmid was designated pGK426-Xe_LDH. Finally, the pGK426-Xe_LDH plasmid was introduced into *HOG1* single and *HOG1* and *CYB2* double disruptants of W303-1B.

### Analysis of the glycerol assimilation ability

The glycerol assimilation ability of *S. cerevisiae* was analyzed by monitoring cell growth on glycerol. The preculture was prepared by culturing *S. cerevisiae* in the MD medium. For the main culture, cells from the preculture washed with MG medium were diluted to an optical density at 660 nm (OD_660_) of 0.05, and then cultured at 30 °C for 168 h. The cells were cultured in 5 mL of MG medium with reciprocal shaking at 150 strokes/min using test tubes. The OD_660_ was measured using a spectrophotometer UV-1280 (Shimadzu Co., Kyoto, Japan).

### L-Lactic acid production assay

Cells of the *HOG1* single and *HOG1* and *CYB2* double disruptants carrying pGK426-Xe_LDH were cultured in 300-mL Erlenmeyer flasks containing 50 mL of modified MG medium with rotary shaking at 200 rpm to prevent sedimentation of the cells. Time courses of OD_660_ and the concentrations of glycerol and L-lactic acid in the culture supernatant were measured. Concentrations of L-lactic acid, ethanol, and glycerol in the culture supernatant were measured using F-kit L-lactate (Roche Diagnostics GmbH, Mannheim, Germany), F-kit ethanol (Roche Diagnostics GmbH), and Glycerol GK assay kit (Megazyme Ltd., Bray, Ireland), respectively.

## Results

### Genome resequencing of the 85_9 mutant strain and identification of the mutation responsible for glycerol assimilation

We previously performed adaptive laboratory evolution of *S. cerevisiae* W303-1B strain to achieve a high growth rate in glycerol as the main carbon source, and the specific growth rate increased by approximately 3-fold after 85 generations (Kawai et al. 2019). The mutant strain 85_9 showed the highest specific growth rate among the strains randomly isolated from the evolved population after 85 generations (Yuzawa et al. 2021). To understand the high glycerol assimilation phenotype of the 85_9 strain, we analyzed the genome sequence of the strain and the evolved population after 85 generations, compared with that of the W303-1B parental strain. We identified mutations in the open reading frame (ORF) of the 85_9 strain, which overlapped in the evolved population after 85 generations. We found that four genes, *HOG1*, *SIR3*, *SSB2*, and *KGD2* had a mutation in their ORFs; a frameshift mutation was found in the ORF of *HOG1*, while missense mutations were found in the ORF of the other genes (Table 1). These mutations in the 85_9 genome were confirmed using Sanger sequencing.

**Table 1:** Mutations in the open reading frame of genes in the 85_9 strain

Chromosome no.	Position	Reference allele (W303-1B)	Mutation allele (85_9)	Gene (gene ID)	Annotation	Mutation type
Chr XII	371,811	CAA	CA	*HOG1* (YLR113W)	Mitogen-activated protein kinase involved in osmoregulation	Frameshift mutation corresponding to Lys65
Chr IV	753,916	T	A	*KGD2* (YDR148C)	2-Oxoglutarate dehydrogenase E2 component	Asn384 (AAT) → Ile (ATT)
Chr XII	1,019,484	A	T	*SIR3* (YLR442C)	Chromatin-silencing protein	Leu923 (CTA) → Gln (CAA)
Chr XIV	252,293	A	G	*SSB2* (YNL209W)	Hsp70 family ATPase	Arg79 (AGA) → Gly (GGA)

**Fig. 1:**
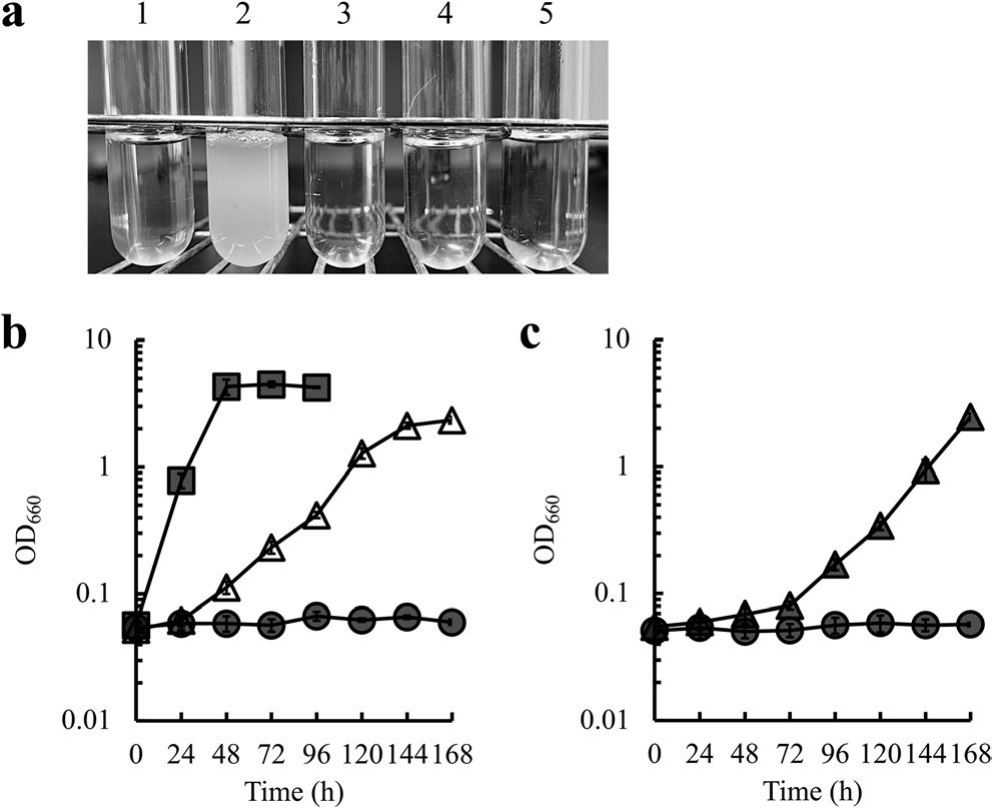
Effect of introducing mutations found in the 85_9 strain into the W303-1B strain on glycerol assimilation. (**a**) Cultures of W303-1B (1) and the strains carrying mutations in *HOG1* (2), *KGD2* (3), *SSB2* (4), and *SIR3* (5) genes found in the 85_9 strain. All strains were cultured in MG medium. The strains carrying mutations of the *HOG1*, *SSB2* and *SIR3* genes were constructed by introducing each mutation into the genome of the W303-1B, while the strain carrying the mutation of the *KGD2* gene was constructed by introducing the plasmid expressing the mutant *KGD2* gene into the *KGD2* disruptant of the W303-1B. (**b**) Time courses of cell growth (OD_660_) of W303-1B (gray circles), *HOG1* frameshift mutant (white triangles), and 85_9 (gray squares) in MG medium. (**c**) Time courses of cell growth of W303-1B (gray circles) and *HOG1* disruptant (gray triangles) in MG medium. In **b** and **c**, results are shown as the mean ± standard deviation of three independent experiments

Next, we determined which mutation(s) caused the high glycerol assimilation ability of the 85_9 strain by introducing each identified mutation into the genome of the parental W303-1B strain and observing cell growth of the mutation-introduced strains on glycerol. Although the mutations in *HOG1*, *SIR3*, and *SSB2* genes were successfully introduced into the genome of the W303-1B strain, the mutation in the *KGD2* gene was not. Therefore, the mutant *KGD2* gene cloned into the plasmid was introduced into the *KGD2*-disrupted W303-1B strain and its growth in glycerol was analyzed. As shown in Fig. 1a, only the strain with a frameshift mutation in the *HOG1* gene grew on glycerol, indicating that the high glycerol assimilation phenotype in the 85_9 strain is caused by a frameshift mutation in the *HOG1* gene.

To investigate whether only the *HOG1* frameshift mutation caused high glycerol assimilation, we compared growth in glycerol of the W303-1B strain carrying the *HOG1* frameshift mutation with that of the 85_9 strain. As shown in Fig. 1b, growth on glycerol was significantly higher in the 85_9 strain than that in the W303-1B strain with a *HOG1* frameshift mutation. This result indicates that other unknown factor(s), in addition to the *HOG1* frameshift mutation, caused high glycerol assimilation in the 85_9 strain.

Furthermore, to determine when these four mutations were introduced through adaptive laboratory evolution, nucleotide sequences of these four genes in the populations obtained from the culture after 3 and 35 generations of adaptive laboratory evolution (Kawai et al. 2019) were analyzed. The *HOG1* frameshift mutation was found in the cells from the populations at 3 and 35 generations, while the other mutations were detected in the cells from the evolved population at 85 generations (data not shown). These results indicate that the *HOG1* frameshift mutation introduced in the first three generations causes an initial increase in cell growth during the adaptive laboratory evolution (Kawai et al. 2019).

### Effect of disruption of genes related to the HOG pathway on glycerol assimilation

Hog1 is a MAPK and is involved in signal transduction in response to osmotic stress via the HOG pathway (Brewster et al. 1993). As the *HOG1* gene mutation in the 85_9 strain was a frameshift mutation (Table 1), we investigated the effect of *HOG1* gene disruption on glycerol assimilation in *S. cerevisiae* W303-1B. As shown in Fig. 1c, disruption of the *HOG1* gene significantly increased glycerol assimilation in *S. cerevisiae*.

**Fig. 2:**
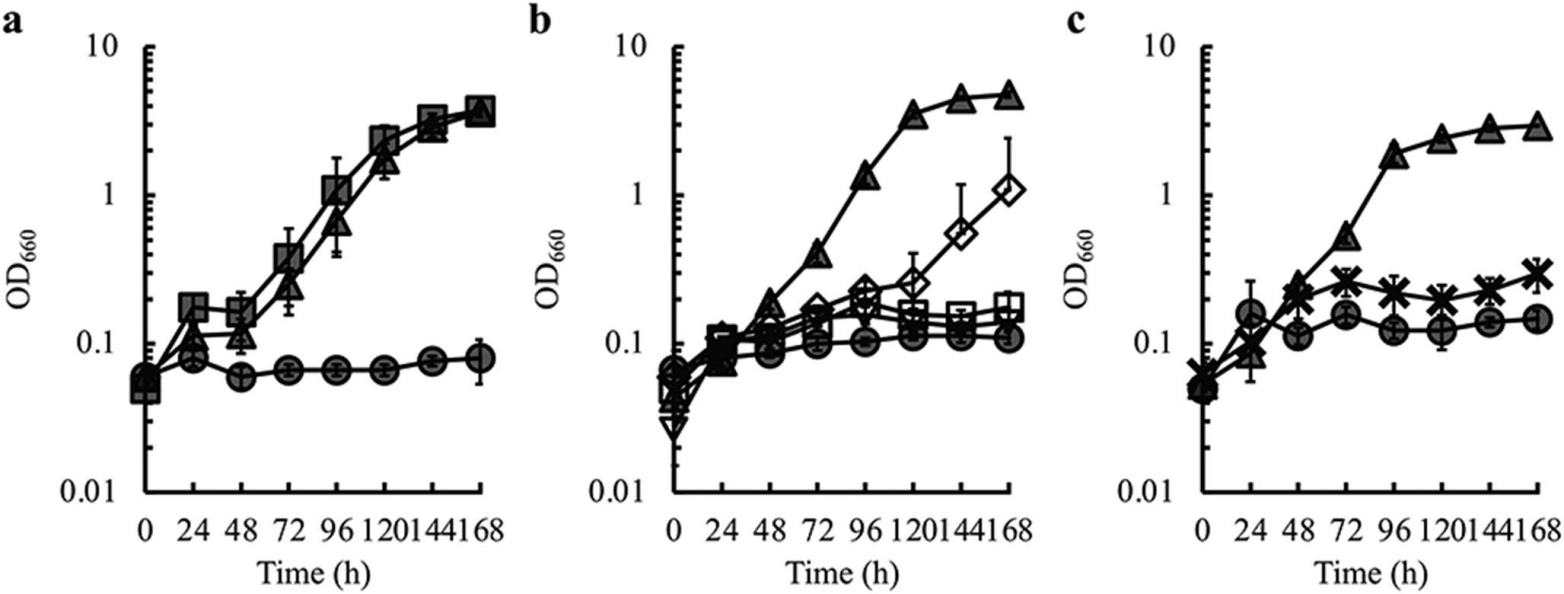
Effect of disruption of genes related to the HOG pathway. (**a**) Effect of disruption of *HOG1* and *PBS2* genes on growth on glycerol. Time courses of cell growth (OD_660_) of W303-1B (gray circles), *HOG1* disruptant (gray triangles), and *PBS2* disruptant (gray squares) in MG medium are shown. (**b**) Effect of disruption of the MAPKKKs in the HOG pathway on growth in glycerol. Time courses of cell growth on W303-1B (gray circles), *HOG1* disruptant (gray triangles), *SSK2 SSK22* double disruptant (white reversed triangles), *STE11* disruptant (white squares), and *SSK2 SSK22 STE11* triple disruptant (white diamonds) in MG medium are shown. (**c**) Effect of triple disruption of *SSK2*, *SSK22*, and *SHO1* genes on growth on glycerol. Time courses of cell growth of W3031-B (gray circles), *HOG1* disruptant (gray triangles), and *SSK2 SSK22 SHO1* triple disruptant (crosses) in MG medium are shown. Results are shown as the mean ± standard deviation from three independent experiments

In the HOG pathway, Hog1 is phosphorylated by Pbs2 MAPKK, and phosphorylated Hog1 phosphorylates its target proteins in the nucleus, including transcription factors and other proteins, to facilitate downstream cellular functions (Brewster et al. 1993). Therefore, we investigated the effect of *PBS2* gene disruption on glycerol assimilation in *S. cerevisiae* and found that the disruption improved cell growth on glycerol (Fig. 2a). These results indicate that loss of function of Hog1 MAPK caused by disruption of *HOG1* and *PBS2* genes improves glycerol assimilation in *S. cerevisiae*.

As described in the Introduction, two individual signal transduction pathways lead to Pbs2 phosphorylation: Ssk2 and Ssk22 MAPKKKs phosphorylate the Pbs2 MAPKK receiving signals from Sln1 protein on the cytoplasmic membrane through Ypd1 and Ssk1, while Ste11 MAPKKK phosphorylates the Pbs2 responding signals by Sho1 protein on the cytoplasmic membrane (Posas and Saito 1997; Posas et al. 1996). Therefore, the effect of disrupting the genes encoding MAPKKKs, which are responsible for Pbs2 phosphorylation on glycerol assimilation, was examined. As shown in Fig. 2b, S. *cerevisiae* did not grow well on glycerol as the main carbon source by disruption of either *STE11*, *SSK2*, or *SSK22* genes, while their triple disruption partially improved growth on glycerol. In addition, triple disruption of *SSK2*, *SSK22*, and *SHO1* did not improve glycerol assimilation (Fig. 2c). These results indicate that inactivation of MAPKKKs in the HOG pathway toward Pbs2 phosphorylation results in partial improvement of glycerol assimilation in *S. cerevisiae*.

### Effect of *RIM15* disruption with *STL1* overexpression on glycerol assimilation in the *HOG1* frameshift mutant

**Fig. 3:**
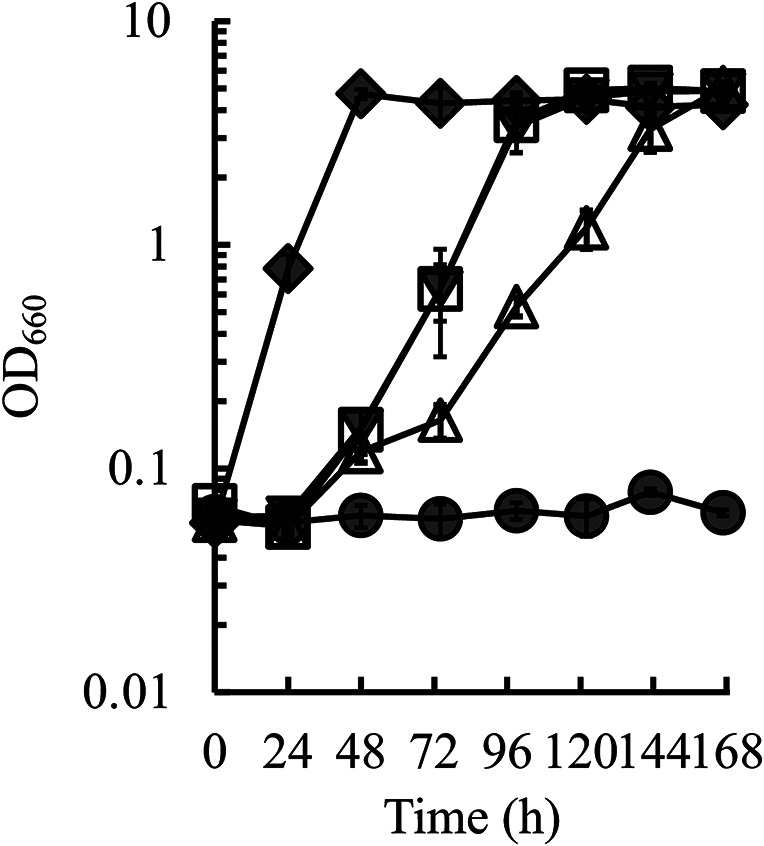
Effect of *RIM15* disruption and *STL1* overexpression on glycerol assimilation in the *HOG1* frameshift mutant. Time courses of cell growth (OD_660_) of W303-1B (gray circles), *HOG1* frameshift mutant (white triangles), *HOG1* frameshift mutant with *RIM15* disruption and *STL1* overexpression (gray reversed triangles), and *RIM15* disruptant with *STL1* overexpression (white squares) and 85_9 (gray diamonds) in MG medium are shown. Results are shown as the mean ± standard deviation from three independent experiments

We previously reported that disruption of the *RIM15* gene encoding a Greatwall protein kinase, together with overexpression of the *STL1* gene encoding a glycerol importer, resulted in increased glycerol assimilation in *S. cerevisiae* (Kawai et al. 2019). Therefore, we examined whether glycerol assimilation in the *HOG1* frameshift mutant was improved by disrupting *RIM15* gene and introducing the *STL1* overexpression plasmid pGK425-*STL1*. As shown in Fig. 3, cell growth of the *HOG1* frameshift mutant was improved by *RIM15* disruption and *STL1* overexpression, but the growth was similar to that of the *RIM15* disruptant overexpressing *STL1*. This result indicates that the synergy of the *HOG1* frameshift mutation with *RIM15* disruption and *STL1* overexpression in glycerol assimilation does not occur.

### Utilization of the *HOG1* disruptant as a host for L-lactic acid production

**Fig. 4:**
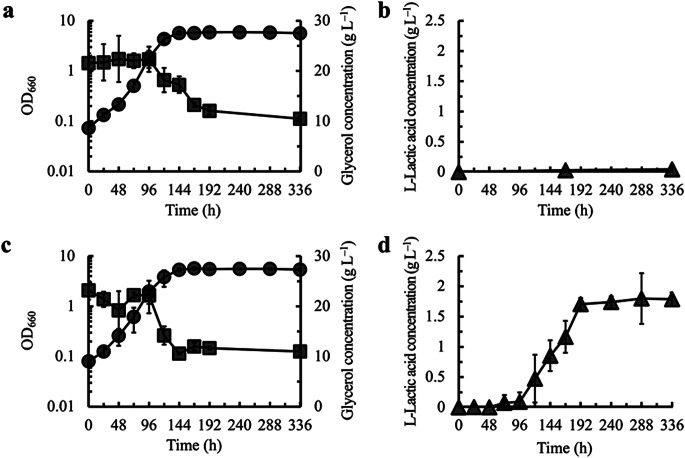
L-Lactic acid production by the *HOG1* disruptant of *S. cerevisiae*. Time courses of cell growth (OD_660_, circles) and glycerol concentration in culture supernatant (squares) and L-lactic acid concentration in culture supernatant (triangles) for the *HOG1* disruptant (**a** and **b**) and *HOG1 CYB2* double disruptant (**c** and **d**) harboring LDH from *X. laevis*. Results are shown as the mean ± standard deviation in three independent experiments

Finally, utilization of the *HOG1* disruptant as a host in the production of valuable materials was attempted. We constructed a recombinant strain of the *HOG1* disruptant to produce L-lactic acid and investigated its L-lactic acid production (Fig. 4). Briefly, the codon-optimized gene encoding LDH from *X. laevis* was cloned under the *PGK1* promoter on the pGK426 vector and introduced into the *HOG1* disruptant.

Before conducting the L-lactic acid production assay, we added uracil, L-leucine, L-histidine, and L-tryptophan, which are required for both strains to grow, to the culture of the *S. cerevisiae* W303-1B strain and its *HOG1* disruptant, and examined the effect on the growth and glycerol assimilation. The final nutrient concentration was two and five times higher (0.15 and 0.38 g L^–1^, respectively) compared with that in the original MG medium (0.076 g L^–1^). As shown in Figs. S2a and S2b, the W303-1B strain did not grow on glycerol or assimilate glycerol, even though uracil, L-leucine, L-histidine, and L-tryptophan were supplemented. In contrast, in the *HOG1* disruptant, when changing concentration of uracil, L-leucine, L-histidine and L-tryptophan in the MG medium, final OD_660_ value reached at 168 h in the presence of 0.38 g L^–1^ of uracil, L-leucine, L-histidine and L-tryptophan was larger than that in the presence of 0.076 g L^–1^ of them and this difference was significant (*p* < 0.05, *t*-test) (Fig. S2c). Moreover, concentration of glycerol remained in the presence of 0.38 g L^–1^ of uracil, L-leucine, L-histidine and L-tryptophan was lower than that in the presence of 0.076 g L^–1^ of them and this difference was also significant (*p* < 0.05, *t*-test)(Fig. S2d). These indicate that glycerol assimilation is improved by additional nutrient supplementation. Therefore, we used modified MG medium, where the concentration of L-leucine, L-histidine, and L-tryptophan was five times higher compared to that in the original MG medium, but uracil was not added because the strain harboring pGK426-Xe_LDH1, which carries the *URA3* gene as a selection marker, was cultured in this study.

L-Lactic acid production by the *HOG1* disruptant harboring LDH from *X. laevis* was examined under microaerobic conditions. As shown in Fig. 4a and b, although glycerol was consumed in the *HOG1* disruptant harboring *X. laevis* LDH, L-lactic acid production was not observed in this strain. Moreover, glycerol consumption stopped after consumption of approximately 8 g L^–1^ for 192 h. As the reason for the lack of L-lactic acid production might be the immediate incorporation of produced L-lactic acid into the cells of the recombinant strain, the reduction of the incorporation of L-lactic acid was attempted. According to Ookubo et al. (2008), disruption of the *CYB2* gene encoding L-lactate cytochrome *c* oxidoreductase, responsible for the reduction of L-lactic acid to pyruvic acid, prevents incorporation of L-lactic acid into the cells due to the decrease in pH in the culture. Therefore, the *CYB2* gene was additionally disrupted in the *HOG1* disruptant harboring LDH from *X. laevis* and L-lactic acid production was examined. As shown in Fig. 4c and Table S2, specific growth rate was not changed by the additional *CYB2* disruption, but specific glycerol consumption rate was significantly increased (*p* < 0.05, *t*-test). Moreover, as shown in Fig. 4d, L-lactic acid production was observed in the LDH-harboring *HOG1* disruptant by *CYB2* gene disruption and the production levels reached approximately 1.8 g L^–1^ at 336 h. Similar to the case of the LDH-harboring *HOG1* disruptant, glycerol consumption stopped after consuming approximately 10 g L^–1^ for 144 h. Ethanol was not produced by any of the recombinant strains (data not shown). In addition, the *CYB2* disruptant harboring the LDH from *X. laevis* could not grow on glycerol (data not shown), revealing that the *CYB2* disruption does not affect glycerol assimilation in *S. cerevisiae*. These results indicate that the *HOG1* disruptant of *S. cerevisiae* can be used for bioproduction from glycerol, but it is necessary to solve the problems regarding stopping glycerol consumption to further increase the production of target materials.

## Discussion

In this study, to investigate the mechanism for improved glycerol assimilation in the *S. cerevisiae* 85_9 strain obtained through adaptive laboratory evolution, we identified a mutation that is related to this phenomenon. We found that the frameshift mutation in the *HOG1* gene causes the increased glycerol assimilation in the 85_9 strain (Fig. 1a and b). However, the growth rate of the *HOG1* frameshift mutant and *HOG1* disruptant was lower than that of the 85_9 strain obtained through adaptive laboratory evolution (Fig. 1b and c), indicating that other factor(s), which is responsible for the high glycerol assimilation phenotype in the 85_9 strain, may be present in addition to the *HOG1* frameshift mutation. In the present study, the mutations in the open reading frame of the genes were focused. However, some mutations in the intergenic regions of the open reading frames should be found as well. Such mutations might be related to improved glycerol assimilation in the 85_9 strain and introduction of the mutations into the *HOG1* frameshift mutant would further improve glycerol assimilation. In addition, ^13^C-metabolic flux analysis of the 85_9 strain revealed that the increase in the flux to the pentose phosphate pathway might be related to improved glycerol assimilation (Yuzawa et al. 2021). Therefore, genetic modification in the *HOG1* frameshift mutant to increase the flux to the pentose phosphate pathway might also be required for further improvement of glycerol assimilation.

In this study, disruption of the *HOG1* disruption improved glycerol assimilation in *S. cerevisiae*, but the growth characteristics of the *HOG1* disruptant seemed to be unstable. The specific growth rates of the *HOG1* disruptant during exponential phase calculated using the data in Figs. 1c and 2a, b and c were similar (0.036 ± 0.000 h^–1^, 0.035 ± 0.001 h^–1^, 0.040 ± 0.002 h^–1^, and 0.037 ± 0.000 h^–1^, respectively), but the lag time of growth shown in Fig. 1c was longer than that shown in Fig. 2. This indicates that there might be less reproducibility of the length of lag time, which corresponds to initial adaptation of the *HOG1* disruptant to the environment where the strain can grow on glycerol as a carbon source. Once the *HOG1* disruptant starts growing after initial adaptation to the condition where it can grow on glycerol, it can exponentially grow well. In addition, rapid adaptation to such environment (i.e. reduction of the lag time) would be required for improving glycerol assimilation in the *HOG1* disruptant.

It was expected that Hog1 MAPK would not be active and glycerol assimilation would increase when the *SSK2*, *SSK22* and *STE11* genes encoding upstream MAPKKKs in the HOG pathway were disrupted. However, this disruption partially improved glycerol assimilation (Fig. 2b). The mechanism for this phenomenon is unclear, but Pbs2 MAPKK seems to be active when grown on glycerol as the carbon source, even though the genes encoding upstream MAPKKKs were disrupted. There might be unidentified protein kinase(s) responsible for phosphorylation of the Pbs2 and/or Hog1, in addition to Ssk2, Ssk22 and Ste11. In the triple disruptant of the *SSK2*, *SSK22* and *STE11* genes, such unidentified protein kinase might moderately activate the Pbs2 and/or Hog1 by phosphorylation and as a result glycerol assimilation in *S. cerevisiae* would partially be improved. In addition, triple disruption of the *SSK2*, *SSK22*, and *SHO1* did not result in improved glycerol assimilation. In the triple disruptant of the *SSK2*, *SSK22*, and *SHO1*, Pbs2 and/or Hog1 might completely be phosphorylated by unidentified protein kinase(s) and as a result, glycerol assimilation would not be improved.

Strucko et al. (2018) reported that mutations in the *HOG1* and *PBS2* genes were found in the evolved populations obtained through adaptive laboratory evolution of *S. cerevisiae* grown on glycerol as the main carbon source. These researchers suggested that strains carrying a mutation in *GUT1* encoding glycerol kinase with that in *HOG1* or *PBS2* show significant growth in glycerol. Mutations in the *HOG1* and *PBS2* genes found in the evolved populations resulted in a loss-of-function phenotype, but they did not examine contribution of the mutations in the *HOG1* and *PBS2* genes to glycerol assimilation in *S. cerevisiae*. In contrast, we found that only the *HOG1* frameshift mutation resulted in significant growth on glycerol. Comparing these studies, it can be concluded that the main cause for significant growth on glycerol is a loss-of-function mutation in the *HOG1* gene. In addition, Kvitek and Sherlock (2013) showed that the signaling pathways, including Ras/cAMP/PKA and HOG pathways, are affected by mutations through adaptive laboratory evolution experiments, resulting in loss of sensitivity to environmental changes. These researchers concluded that the loss of signaling networks is a major strategy for adapting to a constant environment. Therefore, *S. cerevisiae* can grow on glycerol as the carbon source because it does not respond to the environment in which it is grown.

We also demonstrated utilization of the *HOG1* disruptant in *S. cerevisiae* for bioproduction from glycerol. In the present study, the *HOG1* disruptant was used as a host for L-lactic acid production from glycerol. However, since L-lactic acid production was not observed in the LDH-harboring the *HOG1* disruptant, the *CYB2* gene was additionally disrupted in the LDH-expressing *HOG1* disruptant because it was assumed that the L-lactic acid produced might be incorporated into the recombinant cells due to the decrease in the pH of the culture (Ookubo et al. 2008). L-Lactic acid production was observed by additional disruption of the *CYB2* gene. It should be noted that the specific glycerol consumption rate in the LDH-expressing *HOG1 CYB2* double disruptant was higher than that in the LDH-expressing *HOG1* disruptant, whereas their specific growth rates were similar (Table S2); difference in specific glycerol consumption rates between two strains was significant (*p* < 0.05, *t*-test). When *S. cerevisiae* cells consume glycerol, the NADH/NAD^+^ ratio (redox balance) tends to increase (Clomburg and Gonzalez 2013; Merico et al. 2011). Therefore, the increase in the specific glycerol consumption rate might be caused by the increase in NADH oxidation through L-lactic acid production, catalyzed by LDH. Considering these results, it might be useful to determine the compound whose production is related to NADH oxidation as a target for bioproduction from glycerol, using the *HOG1* disruptant of *S. cerevisiae*.

Currently, the mechanism for increased glycerol assimilation by loss of function of the *HOG1* gene is still unclear. It is important to understand the mechanism for further improvement of glycerol assimilation toward bioproduction from glycerol. Moreover, utilization of the *HOG1* disruptant to produce useful material(s) would be encouraged to demonstrate the effectiveness of the *HOG1* disruptant as a bioproduction host.

## Electronic supplementary material

Below is the link to the electronic supplementary material.

Supplementary Material 1

## Data Availability

All data generated or analyzed during this study are included in this article and its supplementary information.
