# Supplementary material for: Loss of function of Hog1 improves glycerol assimilation in Saccharomyces cerevisiae
Source: World J Microbiol Biotechnol. 2023 Jul 21;39(10):255. doi: 10.1007/s11274-023-03696-z (PMC10359374; doi:10.1007/s11274-023-03696-z)
Supplement: Supplementary file 1 — Supplementary Material 1 [file 11274_2023_3696_MOESM1_ESM.pdf]

Supplementary materials

Loss of function of Hog1 improves glycerol assimilation in *Saccharomyces cerevisiae*

Masato Sone[1, †] · Kantawat Navanopparatsakul[1, †] · Shunsuke Takahashi[1] · Chikara Furusawa[2, 3, 4] · Takashi Hirasawa[1, *]

[1]School of Life Science and Technology, Tokyo Institute of Technology, 4259 Nagatsuta-cho, Midori-ku, Yokohama, Kanagawa 226-8501, Japan

[2]Center for Biosystem Dynamics Research, RIKEN, 6-2-3 Furuedai, Suita, Osaka 565-0874, Japan

[3]Universal Biology Institute, The University of Tokyo, 7-3-1 Hongo, Bunkyo-ku, Tokyo 113-0033, Japan

[4]Department of Physics, Graduate School of Science, The University of Tokyo, 7-3-1 Hongo, Bunkyo-ku, Tokyo 113-0033, Japan

[*]Corresponding author

Takashi Hirasawa

thirasawa@bio.titech.ac.jp

ORCID

Takashi Hirasawa 0000-0002-5183-2953

[†]These authors contributed equally to this work.

ATGGCTACCGTTAAGGATAAGTTGATCCATAACGTCGTCAAAGAAGAATCCTTGC
CACAAAACAAGGTTACCATAGTTGGTGTTGGTGCTGTTGGTATGGCTTGTGCTAT
TTCTGTTTTACAAAAGGATTTGGCCGATGAATTGGCCTTGGTTGATGTTATTGAAG
ATAAGTTGAAGGGTGAAATGATGGACTTGCAACATGGTTCTTTGTTCTTGAGAAC
TCCAAAGATCGTTTCCGGTAAGGATTATTCTGTTACCGCTAACTCTAAGTTGGTTG
TTGTTACTGCTGGTGCTAGACAACAAGAAGGTGAATCAAGATTGAACTTGGTCCA
AAGAAACGTCAACATCTTCAAGTTCATCATCCCAAACATCGTCAAGTACTCTCCA
AACTGTACTTTGTTGATTGTCTCCAACCCAGTTGATATCTTGACTTACGTTGCTTG
GAAGATTTCTGGTTTCCCAAAGAACAGAGTTATTGGTTCTGGTTGCAATTTGGATT
CCGCTAGATTCAGATATTTGATGGGTCAAAAGTTCGGTATCCATACCCAATCTTG
TCATGGTTGGGTTATTGGTGAACATGGTGATTCTTCAGTTCCAGTTTGGTCTGGTG
TTAATGTTGCTGGTGTTTCTTTGAAAACCTTGCATCCAGATATTGGTTCCGATGCT
GACAAAGAAAACTGGAAAGAAGTTCACAAGCAAGTTGTTGATTCTGCCTACGAA
GTTATCAAATTGAAGGGTTATACCTCCTGGGCTATTGGTTTGTCTGTTGCTGATTT
GTCCGAATCCATTTTGAAGAACTTGAGAAGAGTTCACCCAATCTCTACTATGGTT
AAGGGTATGTACGGTGTTAACAACGACGTTTTCTTGTCTGTTCCATGTGTTTTGGG
TAACTTGGGTATTACCGATGTTGTCAACATGACTTTGAAGGCTGATGAAGAAGAT
AGATTGAGAAAGTCTGCTGATACCTTGTGGGCCATTCAAAAAGAATTACAATTTT
AA

Fig. S1 Codon-optimized nucleotide sequence of the gene encoding LDH from *X. laevis*

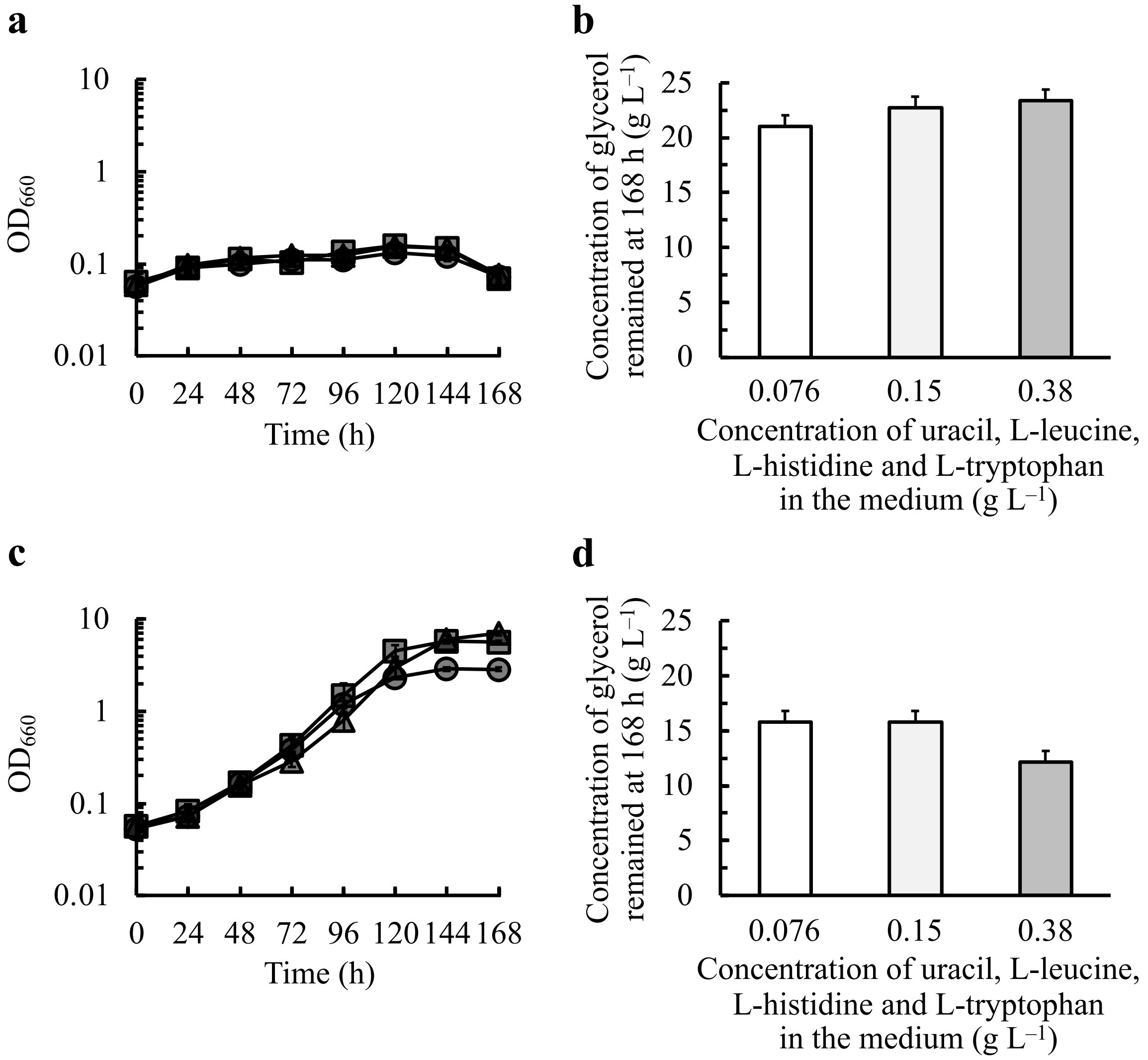

Fig. S2 Effect of additional supplementation of nutrients on glycerol assimilation in the *HOG1* disruptant of *Saccharomyces cerevisiae*

Cell growth on glycerol (**a** and **c**) in the presence of 0.076 (circles), 0.15 (triangles) and 0.38 (squares) g L^{-1} of uracil, L-leucine, L-histidine and L-tryptophan and concentration of glycerol remained in the culture supernatant at 168 h (**b** and **d**) in the W303-1B (**a** and **b**) and its *HOG1* disruptant (**c** and **d**) are shown. In the experiments, initial glycerol concentration was 20 g L^{-1}.

Table S1 PCR primers used in this study

Purpose	Target	Primer sequence (5′→3′)
Amplification of the gene fragment from the 85_9 strain	*HOG1*	TGTATAGTGGAAGAGGAATTTGCG
		GAAGTAAGAATGAGTGGTTAGGGAC
	SIR3	CCTTTCATCACCTTCCTTACAG
		CAACGTTTCCGTGGGTCTG
	SSB2	TTGGGCGACGCAGAGATGAG
		GCCTCAAGAGGGGAAACAG
	KGD2	GCTTCAGGGGAGGAAATATCTAAC
		TCACACAGTAATAGCGGACAAG
Sequence analysis of the gene fragment amplified from the 85_9 strain	*HOG1*	CGGTACAGTTTTCGAGATCAC
	SIR3	GAGACTGCATGTGTACATAGGC
	SSB2	CCAACGAACAAGGTAACAGAG
	KGD2	CAGTCTTCAAGAAGGTAACAGC
Amplification of the fragments of *E. coli mazF*, *S. cerevisiae GAL1* promoter and *S. cerevisiae CYC1* terminator	*E. coli mazF*	AAGGAGAAAAAACTATAATGATGGTAAGCCGATACGTACCCG
		AAGGAAAAGGGGCCTGTTTACTACCCAATCAGTACGTTAATTTTG
	GAL1 promoter	GCGGAGCTCGTACGGATTAGAAGCCGCCGAGCGG
		CATTATAGTTTTTTCTCCTTGACG
	CYC1 terminator	TAAACAGGCCCCTTTTCCTTTGTCG
		GCGGAGCTCGCAAATTAAAGCCTTCGAGCGTCCC
Amplification of *LEU2*-*mazF* and *LEU2* marker cassettes	*HOG1*	AAAAAGGAACAAAGGGAAAACAGGGAAAACTACAACTATCGTATATAATACAGCTGAAGCTTCGTACGC
		ACATCAAAAAGAAGTAAGAATGAGTGGTTAGGGACATTAAAAAAACACGTGCATAGGCCACTAGTGGATCTG
	SIR3	GTACATAGGCATATCTATGGCGGAAGTGAAAATGAATGTTGGTGGCAGCTGAAGCTTCGTACGC
		GGGGTTTAAGAAAGTTGTTTTGTTCTAACAATTGGATTAGCTAAAGCATAGGCCACTAGTGGATCTG
	SSB2	AATGAAAAATATATATATGTGTATAACCTTAACCAGAATGACATCCAGCTGAAGCTTCGTACGC
		TCGTTTTTTCTTTCAAGAAACCAAGAACCAATATCCTCATTAACAGCATAGGCCACTAGTGGATCTG
	KGD2	TACCACATTTGTTACAACCAAAGACACAACTTCAGATAATTATTTAAACACAGCTGAAGCTTCGTACGC
		CGGACAAGAATAATCATGAAATCAGATTGGTATGGGCTGCAAATTTCAAAGCATAGGCCACTAGTGGATCTG
	PBS2	GATACATTATTATATTAAGCAGATCGAGACGTTAATTTCTCAAAGCAGCTGAAGCTTCGTACGC
		GTTGTTATATTCACGTGCCTGTTTGCTTTTATTTGGATATTAACGGCATAGGCCACTAGTGGATCTG

	SSK2	TACTAAAAAGAAGAGAAGCCTTTGCGTAAACTATTTGACAGGCACAAATACAGCTGAAGCTTCGTACGC
		TACATTATATTTTGATTTTACATATAATACAACAAACCTTCTCAACTTAAGCATAGGCCACTAGTGGATCTG
	SSK22	ATGTTGTTTTACTTAGGGTGGCTATAAAAGGTAGTTCCTTGTAGGTGAAACAGCTGAAGCTTCGTACGC
		TACATTACATTATATATCGTAGTATATCATATTTTTAGACGTTGACCACTGCATAGGCCACTAGTGGATCTG
	SHO1	ATAGATCAACGCCATCTTTTCAGAAACACCAAAAATCACGTTTTCAAAATCAGCTGAAGCTTCGTACGC
		TTTATTTTTTTCCTTTGACTCGAGAATCCATGCTATAAGATTGTTAATCAGCATAGGCCACTAGTGGATCTG
	STE11	GACCACTTAATAAAGCTAGTATGATAAGATCACCGGTAGACGAAATATACCAGCTGAAGCTTCGTACGC
		TCGGCCAGAGCACTTTAGTGCCATAAAAAGAATTAATAAGTAGCCCTTTTGCATAGGCCACTAGTGGATCTG
	CYB2	ATACAGTTCCCGCATAGAGAAGAAAGCAAACAAAAGTAGTCAATGAGCTGAAGCTTCGTACGC
		TAAATAAAAAGTTTTTAAAGTAGCCTTAAAGCTAGGCTATAATCACATAGGCCACTAGTGGATCTG
Amplification of the target region to confirm the mutation introduced into the W303-1B strain	*HOG1*	TTCGGCATTTTGACATACAG
		CCAGTTTTACAAGAAAATCC
	SIR3	GCAATTCATTCTTGTCCACC
		TTATCCTCAAGGCCTGCAAC
	SSB2	TGGATTTATAGATTATGCGG
		CATGGTTGTTTTATGCAGCC
Confirmation of gene deletion	*HOG1*	TGTATAGTGGAAGAGGAATTTGCG
		GAAGTAAGAATGAGTGGTTAGGGAC
	KGD2	GCTTCAGGGGAGGAAATATCTAAC
		TCACACAGTAATAGCGGACAAG
	PBS2	GGGTACACGTTTCACAGAACTAC
		AGGTTTTTTTGTATTCGCCGC
	SSK2	TAACTTTTGTGTGAGCTCCG
		ATCCACCTCGACTAGACTTC
	SSK22	AGCGATGCCAGGAACAATAG
		GTTAGTGTGTGTTTATCGAGG
	SHO1	TGAGCCTCGTTTTCAGTGAG
		GAGAATATTTCAGTGAAGCGG
	STE11	TAAGGTGTAAAGCTTTCGCCG
		GGCCAGAGCACTTTAGTGC
	CYB2	TCCTCCTGTTCGAGAGAACTACGAC
		TTCTCTGAACAGCACAATGTCCTCG

Table S2 Specific rates of cell growth, glycerol consumption and L-lactic acid production in the *HOG1* single and *HOG1 CYB2* double disruptants of *S. cerevisiae* harboring LDH from *Xenopus laevis*

	LDH-harboring *HOG1* disruptant	LDH-harboring *HOG1 CYB2* double disruptant
Specific growth rate (h^{-1}) [48–120 h]	0.043±0.003	0.038±0.000
Specific glycerol consumption rate (g g-dry $cell^{-1}$ h^{-1}) [0–144 h]	0.080±0.007	0.182±0.056
Specific L-lactic acid production rate (g g-dry $cell^{-1}$ h^{-1}) [0–192 h]	–	0.012±0.003

For calculation, the data shown in Fig. 3 was used and mean ± standard deviation is shown. In addition, cell concentration was calculated by the following equation:

Cell concentration (g-dry cell L^{-1}) = $0.3 \times OD_{660}$.
